# Prevalence of Hypovitaminosis D Among Patients With Type 2 Diabetes Mellitus: Evidence From a Diabetes Clinic in Durgapur, West Bengal

**DOI:** 10.7759/cureus.108923

**Published:** 2026-05-15

**Authors:** Sutanuka Saha, Piush Pandey, Indradeep Ganguly, Rakesh Kumar, Soumit Roy, Raghunath Misra, Sayanti Bandyopadhyay, Ankita Banerjee, Anirban Mondal, Subarna Sinha Mahapatra

**Affiliations:** 1 Community Medicine, IQ City Medical College and Hospital, Durgapur, IND; 2 Community Medicine, Deben Mahata Government Medical College and Hospital, Durgapur, IND

**Keywords:** diabetic nephropathy, diabetic peripheral neuropathy, diabetic retinopathy, hypovitaminosis d, insulin resistance, microvascular complications, obesity, pwd

## Abstract

Background: Type 2 diabetes mellitus (T2DM) is one of the leading causes of morbidity and mortality worldwide. Studies have shown that vitamin D is associated with obesity and insulin resistance. However, hypovitaminosis D may represent a largely hidden burden, as many cases remain undiagnosed.

Objectives: This study aimed to determine the proportion and predictors of hypovitaminosis D among people with T2DM attending a specialty clinic in Durgapur, West Bengal, and to elucidate the association between hypovitaminosis D and microvascular complications of T2DM.

Materials and methods: An analytical cross-sectional study was conducted over a period of two months among 89 patients with T2DM attending a diabetic specialty clinic, using a predesigned, pretested structured schedule. Clinical histories were obtained, and anthropometric measurements were performed in accordance with standard operating procedures. Vitamin D levels and HbA1c were measured through blood investigations. Data were analyzed using SPSS Statistics version 26.0 (IBM Corp. Released 2019. IBM SPSS Statistics for Windows, Version 26.0. Armonk, NY: IBM Corp.). Pearson’s chi-square test and univariable and multivariable logistic regression analyses were performed to identify predictors of hypovitaminosis D.

Results: Hypovitaminosis D was present in 71 (79.8%) study participants. The majority of participants were female (62.9%), and most were overweight and/or obese (80.9%). Age greater than 45 years, female gender, higher BMI (overweight or obese), and uncontrolled T2DM (HbA1c ≥7) were identified as risk factors for hypovitaminosis D. Age ≥45 years, female gender, and uncontrolled T2DM were found to be significant predictors of hypovitaminosis D. A significant association was observed between hypovitaminosis D and microvascular complications of T2DM.

Conclusions: The prevalence of hypovitaminosis D among patients with T2DM was high. Increasing age, female gender, poor glycemic control, and microvascular complications of T2DM were significantly associated with hypovitaminosis D. Further research is needed to improve understanding of the predictors of hypovitaminosis D among patients with T2DM.

## Introduction

Type 2 diabetes mellitus (T2DM) affects more than 300 million people worldwide and is associated with significant morbidity and mortality rates [1]. India is often referred to as the “Diabetes Capital of the World” [2].

Vitamin D is traditionally associated with calcium and phosphorus homeostasis and the regulation of bone health. However, recent evidence from various lines of research indicates that vitamin D also plays an indirect role in several aspects of human health, including cancer, infections, respiratory disorders, and cardiovascular diseases [1,3,4].

Patients with T2DM are at increased risk of developing microvascular complications, including diabetic retinopathy, diabetic nephropathy, and diabetic neuropathy [5]. Common risk factors for diabetic microvascular complications include poor glycemic control, increasing age, physical inactivity, longer duration of diabetes, and substance abuse. Glycated hemoglobin (HbA1c) is an important indicator of glycemic control and reflects cumulative blood glucose levels over the preceding three months [6,7].

Hypovitaminosis D has emerged as a potential contributor to the development of both T1DM and T2DM. The association between hypovitaminosis D and glucose intolerance was first reported more than 20 years ago by Pietschmann et al., who demonstrated that serum 25-hydroxyvitamin D (25(OH)D) levels were decreased in patients with T2DM [4,8]. 25(OH)D levels between 30 and 50 ng/mL are considered optimal, whereas levels between 21 and 29 ng/mL indicate vitamin D insufficiency [9,10].

This study aimed to determine the proportion and predictors of hypovitaminosis D among people with T2DM attending a specialty clinic in Durgapur, West Bengal, and to elucidate the association between hypovitaminosis D and microvascular complications of T2DM.

## Materials and methods

Study design and sampling

This was an analytical, clinic-based, cross-sectional study conducted from January 2025 to March 2025. The sample size was calculated using Cochran’s formula:

$\text{Sample size (n)} = Z_{(1-\alpha/2)}^{2} \cdot \frac{P(1-P)}{L^{2}}$

where $Z_{(1-\alpha/2)} = 1.96$ at a 95% confidence interval, the absolute error (L) was 10%, and P represented the prevalence of hypovitaminosis D from a previous study, which was 74.18%. A nonresponse rate of 20% was considered, and the calculated minimum sample size was 89 participants [9]. Simple random sampling was performed using the outpatient department register as the sampling frame.

Data collection method and standard operating protocol

Adults with T2DM attending Kumar’s Diabetes Clinic outpatient department in Durgapur were evaluated, and written informed consent was obtained from all participants. People aged 18 years or older with T2DM diagnosed for more than six months were included in the study. Patients without consent, with neurological disorders, had a history of cerebrovascular accident, were receiving steroid therapy, had liver disease, had chronic kidney disease on hemodialysis, or had any condition affecting intestinal absorption of vitamin D were excluded from the study.

Clinicosocial data collected included age, gender, substance abuse, duration of T2DM, and type of antidiabetic medication used. Height was measured to the nearest 0.1 cm using a Prestige floor-mounted stadiometer, whose vertical alignment was checked with a spirit level before measurements began each day. Weight was recorded to the nearest 100 g using an Equinox EB-9300 digital weighing scale (Equinox Systems, Fort Lauderdale, FL, USA), which was zero-calibrated before every reading. BMI was calculated as weight in kilograms divided by height in meters squared:

$\mathrm{BMI} = \frac{\text{Weight (kg)}}{\text{Height (m)}^{2}}$

Laboratory investigations included HbA1c, urea, creatinine, and serum vitamin D levels. Nephropathy was evaluated using the urinary albumin-to-creatinine ratio (uACR) and estimated glomerular filtration rate (eGFR). The uACR was assessed using a random spot urine sample and measured by radioimmunoassay. The eGFR was calculated using the Modification of Diet in Renal Disease equation:

$\mathrm{eGFR} = 186 \times (\text{Serum Creatinine})^{-1.154} \times (\mathrm{Age})^{-0.203} \times (0.742 \ \text{if female})$

Nephropathy was defined as a uACR >30 mg/g, eGFR ≤60 ml/min/1.73 m², or a past history of diabetic nephropathy [11,12]. Chemiluminescence immunoassay was used to measure 25(OH)D levels. Vitamin D levels <30 ng/mL were considered low, whereas levels ≥30 ng/mL were considered normal [5].

Diabetic retinopathy screening was performed using the noninvasive AI-based instrument REVELO with deep learning-powered software (Revelo, Miami, FL, USA) [13,14]. Screening for diabetic neuropathy included assessment of symptoms such as foot drop, aching pain, numbness, tingling sensation, ulcers, and infections in the legs or feet during the previous 2 weeks. Additionally, the Semmes-Weinstein 5.07 (10 g) monofilament examination (SWME) was performed according to guidelines for diabetic and neuropathic ulcers to assess loss of protective sensation in the extremities. Loss of sensation at any site was considered abnormal. The presence of at least one positive symptom of neuropathy and/or one abnormal SWME finding was considered indicative of diabetic peripheral neuropathy [15].

Ethical considerations

Ethical approval for the study was obtained from the Institutional Ethics Committee of IQ City Medical College and Hospital prior to data collection (IQMC/IEC-21/LTR/1, dated 20th January 2025). The study adhered strictly to the ethical principles of autonomy, beneficence, informed consent, confidentiality, non-maleficence, and justice.

Statistical analysis

Data were entered into Excel (Microsoft Corp., Redmond, WA, USA) and checked twice for accuracy and consistency. Statistical analysis was performed using SPSS Statistics version 26.0 (IBM Corp. Released 2019. IBM SPSS Statistics for Windows, Version 26.0. Armonk, NY: IBM Corp.) and Jamovi (Retrieved from https://www.jamovi.org). Descriptive statistics were presented as frequencies and percentages for categorical variables, and as mean ± standard deviation or median (interquartile range) for continuous variables. Normality of data was assessed using Q-Q plots, the Shapiro-Wilk test, and the Kolmogorov-Smirnov test. The chi-square test, along with univariate and multivariable logistic regression analyses, was performed to assess associations between variables and hypovitaminosis D and to identify its predictors. The chi-square test was also used to assess the association between hypovitaminosis D and other microvascular complications.

## Results

The total number of study participants was 89. Hypovitaminosis D was present in 71 (79.8%) participants. Age and serum vitamin D levels were not normally distributed, as indicated by a significant Kolmogorov-Smirnov test (P < 0.05), a positively skewed distribution (mode < median < mean), and characteristic Q-Q plots. The median age of the participants was 51 years (IQR 44.5-57), while the median serum vitamin D level was 20 ng/mL (IQR 12.57-27.39). More than half of the participants were female (56, 62.9%).

HbA1c and duration of T2DM were approximately normally distributed, as shown by an insignificant Kolmogorov-Smirnov test (P > 0.05), a relatively symmetric distribution, and Q-Q plot patterns. The mean HbA1c was 9.48 ± 2.58, and the mean duration of diabetes was 9.2 ± 7.63 years.

The chi-square test showed a significant association between hypovitaminosis D and age (χ² = 29.5, df = 1, P = 0.001), gender (χ² = 8.4, df = 1, P = 0.004), BMI (χ² = 13.9, df = 1, P = 0.001), and HbA1c level (χ² = 12.7, df = 1, P = 0.001) (Table 1).

**Table 1:** Association between hypovitaminosis D and clinicosocial characteristic of study participants (n = 89) P-values in bold indicate statistical significance (P < 0.05). χ2*: chi-square, df: degrees of freedom, HbA1c: glycated hemoglobin, BMI: body mass index

Variables	Hypovitaminosis D absent (n = 18) n (%)	Hypovitaminosis D present (n = 71) n (%)	χ2* (df)	P-value
Age:				
<45	13 (61.9)	8 (38.0)	29.594 (1)	0.001
≥45	5 (7.3)	63 (92.6)		
Gender:				
Male	12 (36.3)	21 (63.6)	8.467 (1)	0.004
Female	6 (10.7)	50 (89.2)		
BMI:				
Underweight and normal	9 (52.9)	8 (47.0)	13.941 (1)	0.001
Overweight and obese	9 (12.5)	63 (87.5)		
Duration:				
<10 years	14 (24.1)	44 (75.8)	2.513 (2)	0.285
10-20 years	4 (16.6)	20 (83.3)		
>20 years	0	7 (100)		
HbA1c:				
<7	10 (47.6)	11 (52.3)	12.784 (1)	0.001
≥7	8 (11.7)	60 (88.2)		

Univariate logistic regression analysis showed that age >45 years was significantly associated with hypovitaminosis D (OR = 20.475, 95% CI: 5.768-72.68, P = 0.001). Female gender was also a significant risk factor (OR = 4.762, 95% CI: 1.578-14.371, P = 0.006). Higher BMI (overweight and obesity) was associated with increased odds of hypovitaminosis D (OR = 7.875, 95% CI: 2.417-25.654, P = 0.001). Similarly, uncontrolled diabetes mellitus (HbA1c ≥7) was significantly associated with hypovitaminosis D (OR = 6.818, 95% CI: 2.202-21.113, P = 0.001) (Table 2).

**Table 2:** Univariate binary logistic regression showing the risk factors of hypovitaminosis D in patients with T2DM (n = 89) P-values in bold indicate statistical significance (P < 0.05). T2DM: type 2 diabetes mellitus, OR: odds ratio, CI: confidence interval, HbA1c: glycated hemoglobin, BMI: body mass index

Variables	Sub-categories	OR, 95% CI	P-value
Age	<45	1	0.001
	≥45	20.475 (5.768, 72.68)	
Gender	Male	1	0.006
	Female	4.762 (1.578, 14.371)	
BMI	Underweight and normal	1	0.001
	Overweight and obese	7.875, (2.417, 25.654)	
Duration	<10 years	1	-
	10-20 years	1.591 (0.465, 5.446)	0.460
	>20 years	514014722.7 (0.000, NA)	0.999
HbA1c	<7	1	0.001
	≥7	6.818, (2.202, 21.113)	

Multivariable logistic regression analysis revealed that age ≥45 years (adjusted OR = 15.911, 95% CI: 3.425-73.906, P = 0.001), female gender (adjusted OR = 10.198, 95% CI: 1.628-63.872, P = 0.013), and uncontrolled T2DM (HbA1c ≥7) (adjusted OR = 11.809, 95% CI: 1.744-79.972, P = 0.011) were significant independent predictors of hypovitaminosis D (Table 3).

**Table 3:** Multivariable logistic regression showing predictors of hypovitaminosis D among the study participants (n = 89) P-values in bold indicate statistical significance (P < 0.05). CI: confidence interval (lower limit, upper limit), aOR: adjusted odds ratio, CI: confidence interval, HbA1c: glycated hemoglobin, BMI: body mass index

Variables	Sub-categories	aOR, 95% CI	P-value
Age	<45	1	<0.001
	≥45	15.911 (3.425, 73.906)	
Gender	Male	1	0.013
	Female	10.198 (1.628, 63.872)	
BMI	Underweight and normal	1	0.543
	Overweight and obese	1.698 (0.308, 69.483)	
HbA1c	<7	1	0.011
	≥7	11.809 (1.744, 79.972)	

Duration of T2DM was not statistically significant in the univariate model; therefore, it was not included in the multivariable logistic regression analysis.

The overall model fit was good, as indicated by a significant Omnibus test (P < 0.0001) and a non-significant Hosmer-Lemeshow test (P = 0.780). The proportion of variance in the dependent variable explained by the model was moderate to substantial, with a Cox and Snell R² of 0.419 and a Nagelkerke R² of 0.660, indicating that the model accounted for approximately 41.9% to 66.0% of the variability in hypovitaminosis D.

**Figure 1:**
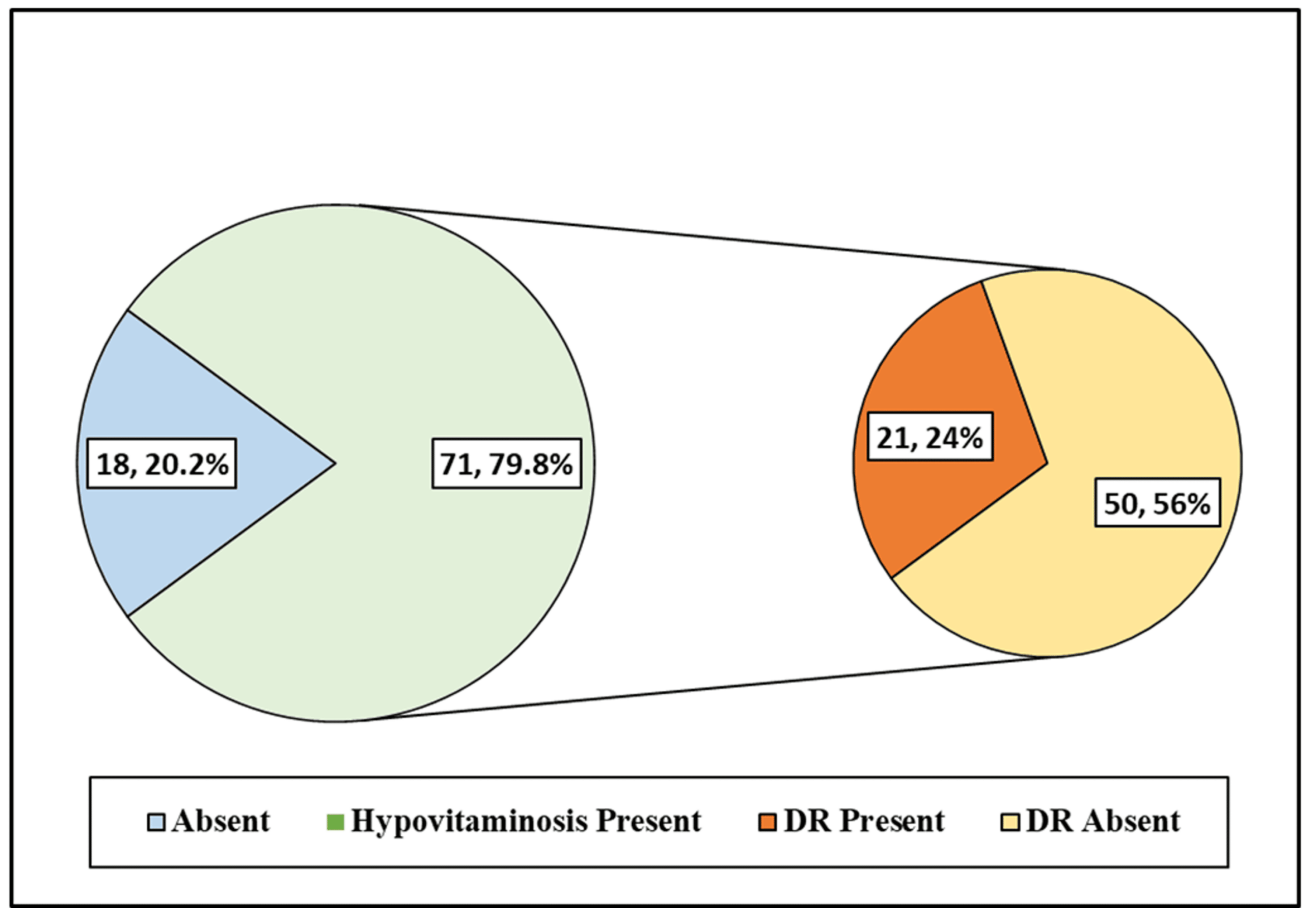
Pie-in-pie diagram showing distribution of study participants based on their hypovitaminosis D status and DR status (n = 89) DR: diabetic retinopathy

Regarding the association between hypovitaminosis D and microvascular complications of T2DM, nearly four-fifths (79.8%) of participants had hypovitaminosis D. Among those with hypovitaminosis D, approximately one-fourth (24%) had co-existing diabetic retinopathy (Figure 1).

**Figure 2:**
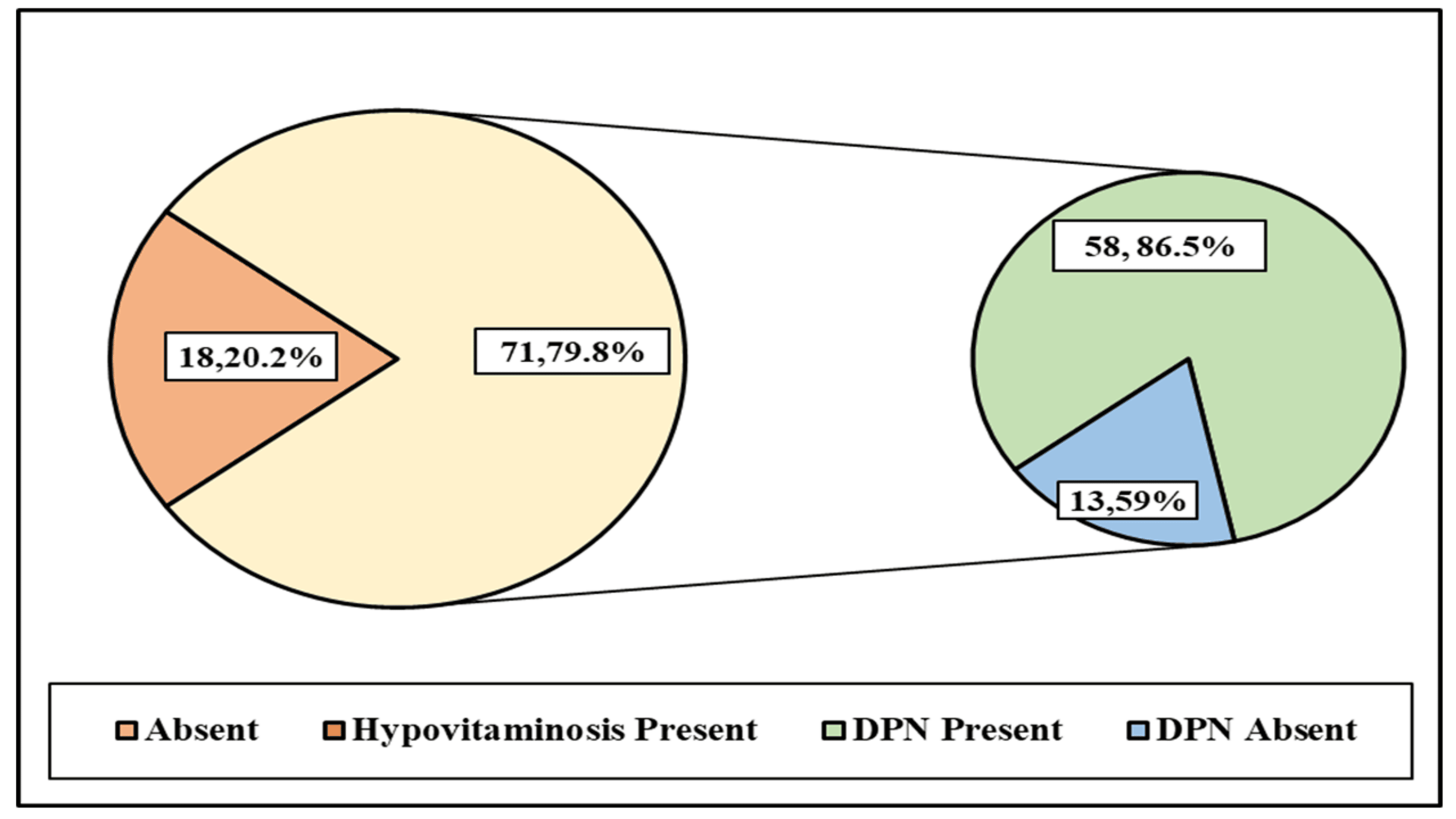
Pie-in-pie diagram showing distribution of study participants based on their hypovitaminosis D status and DPN status (n = 89) DPN: diabetic peripheral neuropathy

Among participants with hypovitaminosis D, the majority (86.5%) had diabetic peripheral neuropathy (Figure 2).

**Figure 3:**
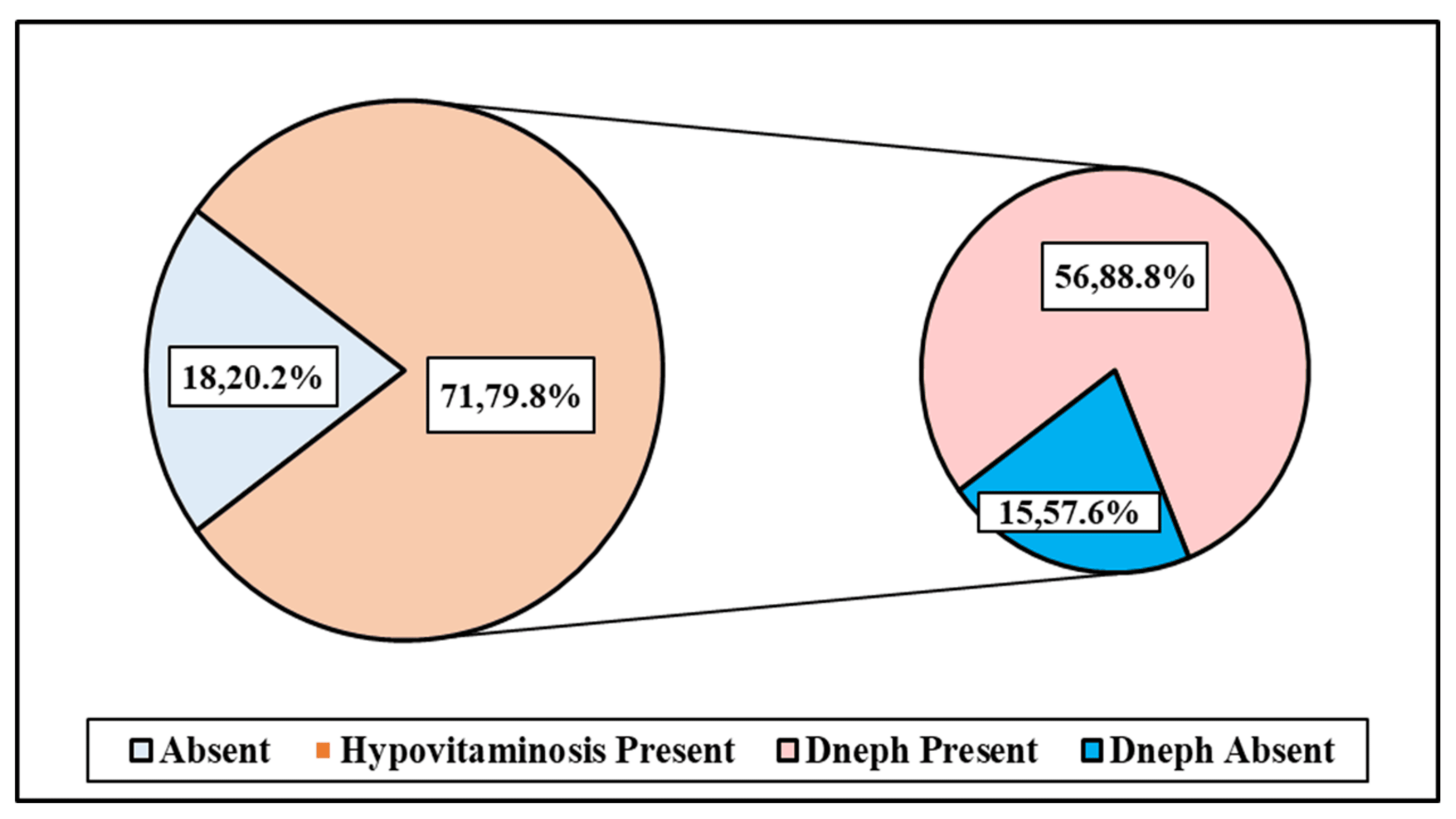
Pie-in-pie diagram showing distribution of study participants based on their hypovitaminosis D status and Dneph status (n = 89) Dneph: diabetic nephropathy

Among participants with hypovitaminosis D, the majority (88.8%) had diabetic nephropathy (Figure 3).

A statistically significant association was observed between hypovitaminosis D and microvascular complications of T2DM, as shown in Table 4.

**Table 4:** Association between hypovitaminosis D and microvascular complications (DR, DNeph, DPN) (n = 89) P-values in bold indicate statistical significance (P < 0.05). χ2*: chi-square, df: degrees of freedom, DR: diabetic retinopathy, DNeph: diabetic nephropathy, DPN: diabetic peripheral neuropathy

Microvascular complication		Hypovitaminosis D present (n = 71) n (%)	Hypovitaminosis D absent (n = 18) n (%)	χ2* (df)	P-value
DR	Present	21	1	4.453 (1)	0.035
	Absent	50	17		
DNeph	Present	58	9	7.749 (1)	0.005
	Absent	13	9		
DPN	Present	56	7	11.102 (1)	0.001
	Absent	15	11		

## Discussion

The present study found the proportion of hypovitaminosis D to be 79.8%. This finding is strikingly comparable to that of Vijay et al., who reported a prevalence of 74.18% among patients with T2DM across India [9,16]. Similarly, Palazhy et al. reported a high prevalence of hypovitaminosis D (71.4%) among T2DM subjects in South India [17]. This alarmingly high prevalence is not surprising in the Indian context, despite the country’s tropical location and year-round sunshine. Ritu and Gupta highlighted that socioreligious and cultural practices, limited vitamin D fortification in the diet, and lifestyle-related barriers to sun exposure contribute to this paradoxically high prevalence, which ranges from 70% to 100% across population subgroups in India [6,18,19]. A community-based study from West Bengal by Srimani et al. further demonstrated that reduced sun exposure and diabetes were significant predictors of vitamin D insufficiency among urban women, supporting the relevance of our findings in this setting [8]. In addition, studies by Pietschmann et al., Ashinne et al., Bajaj et al., Chen et al., and Hong et al. have reported associations between hypovitaminosis D and microvascular complications of T2DM [4,18-21].

In the present study, age ≥45 years was identified as a predictor of hypovitaminosis D in both univariate (OR = 20.47, P < 0.001) and multivariable (aOR = 15.91, P < 0.001) models. This finding is consistent with studies by Ahmadieh et al. and Vijay et al., both of which reported increasing age as a predictor of hypovitaminosis D among people with T2DM [1,9]. This association may be attributed to reduced cutaneous synthesis of vitamin D, decreased renal 1α-hydroxylase activity, and reduced sun exposure with advancing age. These factors, when compounded by the metabolic derangements of long-standing T2DM, may explain the observed association [6,15,16]. Notably, the median age of our study population was 51 years, placing most participants in a higher-risk age group.

Female gender was also identified as a predictor of hypovitaminosis D in both univariate (OR = 4.76, P = 0.006) and multivariable analyses (aOR = 10.20, P = 0.013). This is consistent with the findings of Vijay et al. [9]. Similar observations have been reported by Jayashri et al., who attribute the higher prevalence among women to traditional clothing limiting sun exposure, predominantly indoor lifestyles, lower physical activity, postmenopausal status, and lower intake of vitamin D-rich foods such as fatty fish and fortified dairy products [6,7]. However, this finding is not universally consistent; another study reported higher odds of vitamin D deficiency among men (OR = 0.66 for women), which was attributed to greater occupational sun exposure among males in urban settings [22,23].

In the present study, overweight and obesity (BMI ≥25 kg/m²) were significantly associated with hypovitaminosis D in univariate analysis (OR = 7.88, P = 0.001). However, this association was no longer significant in the multivariable model (aOR = 1.70, P = 0.543), suggesting confounding by age, gender, and glycemic control. The observed univariate association is biologically plausible and well-documented. Obesity was associated with lower circulating 25(OH)D levels due to volumetric dilution and sequestration of fat-soluble vitamin D in adipose tissue, thereby reducing its bioavailability [24-26]. Vijay et al. similarly identified higher BMI as a factor associated with vitamin D deficiency [9], while Agarwal and Sharma reported a similar association among postmenopausal women in India [5]. The loss of significance in the multivariable model suggests that glycemic control may be a stronger determinant of vitamin D status in this population.

Uncontrolled T2DM (HbA1c ≥7%) was a significant predictor of hypovitaminosis D in both univariate (OR = 6.82, P = 0.001) and multivariable analyses (aOR = 11.81, P = 0.011), making it one of the strongest independent predictors after age. The mean HbA1c in this study was 9.48 ± 2.58, indicating that diabetes was poorly controlled in most participants. This is consistent with Ahmadieh et al., who demonstrated a significant association between vitamin D deficiency and poor glycemic control [1]. A study from urban India also reported higher odds of vitamin D deficiency in patients with HbA1c >8% compared to those with better glycemic control [5]. Mechanistically, vitamin D was found to influence pancreatic β-cell function by activating the vitamin D receptor (VDR), modulating inflammatory cytokines, and enhancing insulin sensitivity. Conversely, persistent hyperglycemia might impair renal conversion of 25(OH)D to its active form, thereby creating a bidirectional relationship between poor glycemic control and vitamin D deficiency [1,10,14].

Duration of T2DM was not statistically significant in the univariate model and was therefore not included in the multivariable analysis. Although some studies suggest that longer duration of diabetes contributes to progressive vitamin D deficiency through cumulative metabolic and renal effects, this relationship was not observed in the present study [4]. Vijay et al. similarly reported that duration of diabetes was not a consistent independent predictor, suggesting that age and glycemic control may be more proximal determinants [9].

Hypovitaminosis D and microvascular complications

The present study demonstrated a significant association between hypovitaminosis D and all three microvascular complications of T2DM: diabetic retinopathy, diabetic nephropathy, and diabetic peripheral neuropathy. These findings are consistent with those of Bajaj et al., who reported significant associations between lower vitamin D levels and microvascular complications in Indian patients with T2DM [19]. Chen et al. further demonstrated that low vitamin D levels and VDR polymorphisms independently increase the risk of microvascular complications [20]. Regarding diabetic nephropathy, Hong et al. reported a strong association between vitamin D deficiency and diabetic nephropathy. They proposed that vitamin D might exert renoprotective effects by suppressing the renin-angiotensin-aldosterone system and reducing glomerular injury [21]. Similarly, Ashinne et al. found a significant association between low vitamin D levels and diabetic retinopathy [18]. Pietschmann et al. also highlighted metabolic derangements in T2DM that may link hypovitaminosis D with microvascular damage [4].

Strengths and limitations

The use of multivariable logistic regression enabled adjustment for potential confounders and the identification of independent predictors of hypovitaminosis D. However, because this was a hospital-based cross-sectional study, the findings may not be fully generalizable to the broader community. The relatively high absolute error (10%) used in sample size calculation resulted in a modest sample size. Berksonian bias may also have influenced the findings. Additionally, the cross-sectional design precludes causal inference regarding the relationship between vitamin D status and microvascular complications. Biochemical markers such as parathyroid hormone, serum calcium, phosphate, and inflammatory markers were not assessed, which limits mechanistic interpretation. Further multicentric studies with larger sample sizes are recommended to improve external validity and confirm these findings.

## Conclusions

An alarmingly high proportion of people with T2DM, 71 (79.8%), attending the clinic were found to have hypovitaminosis D, consistent with broader trends across India. The burden was most pronounced among older women and individuals with poor glycemic control, with age emerging as the strongest predictor in this study. Significant associations were observed between hypovitaminosis D and diabetic nephropathy, peripheral neuropathy, and retinopathy, although none remained independently significant in multivariable models. Overall, patients with T2DM, particularly older women with inadequate glycemic control, represent a high-priority group for routine vitamin D screening and timely intervention.

Given the established roles of vitamin D in insulin secretion, pancreatic β-cell function, and the modulation of microvascular injury, these findings support the incorporation of routine vitamin D screening into standard care for patients with T2DM in diabetes clinics across West Bengal and similar urban Indian settings. Public health strategies promoting safe sun exposure, dietary optimization using locally available vitamin D-rich foods such as fish, and targeted supplementation for high-risk groups may serve as useful adjuncts to standard diabetes management. These integrated approaches may help reduce long-term microvascular complications and overall disease burden, although larger prospective studies are needed to confirm causality and clinical benefit.
